# Development of multiplex RT‐ddPCR assays for detection of SARS‐CoV‐2 and other common respiratory virus infections

**DOI:** 10.1111/irv.13084

**Published:** 2022-12-14

**Authors:** Nathaniel K. C. Leong, Haogao Gu, Daisy Y. M. Ng, Lydia D. J. Chang, Pavithra Krishnan, Samuel S. M. Cheng, Malik Peiris, Leo L. M. Poon

**Affiliations:** ^1^ School of Public Health, LKS Faculty of Medicine The University of Hong Kong Hong Kong; ^2^ Centre for Immunology and Infection Hong Kong Science and Technology Park Hong Kong; ^3^ HKU‐Pasteur Research Pole, School of Public Health, LKS Faculty of Medicine The University of Hong Kong Hong Kong

**Keywords:** co‐infection, COVID‐19, human respiratory viruses, RT‐ddPCR, SARS‐CoV‐2

## Abstract

**Background:**

Measures for mitigation of Coronavirus Disease 2019 (COVID‐19) were set to reduce the spread of Severe Acute Respiratory Syndrome Coronavirus 2 (SARS‐CoV‐2). SARS‐CoV‐2 and other respiratory viruses share similar transmission routes and some common clinical manifestations. Co‐circulation of SARS‐CoV‐2 and other common respiratory viruses is imminent. Therefore, development of multiplex assays for detecting these respiratory viruses is essential for being prepared for future outbreaks of respiratory viruses.

**Methods:**

A panel of three reverse transcription droplet digital PCR (RT‐ddPCR) assays were developed to detect 15 different human respiratory viruses. Evaluations of its performance were demonstrated. A total of 100 local and 98 imported COVID‐19 cases in Hong Kong were screened for co‐infection with other common respiratory viruses.

**Results:**

All detected viral targets showed distinct signal clusters using the multiplex RT‐ddPCR assays. These assays have a broad range of linearity and good intra‐/inter‐assay reproducibility for each target. The lower limits of quantification for all targets were ≤46 copies per reaction. Six imported cases of COVID‐19 were found to be co‐infected with other respiratory viruses, whereas no local case of co‐infection was observed.

**Conclusions:**

The multiplex RT‐ddPCR assays were demonstrated to be useful for screening of respiratory virus co‐infections. The strict preventive measures applied in Hong Kong may be effective in limiting the circulation of other human respiratory viruses. The multiplex assays developed in this study can achieve a robust detection method for clinical and research purposes.

## 1 INTRODUCTION

Hong Kong and Mainland China follow the ‘dynamic‐zero’ strategy to control Coronavirus Disease 2019 (COVID‐19).^1^ The practice of social distancing, mandatory mask‐wearing and extensive screening of travellers/local people has been used to reduce Severe Acute Respiratory Syndrome Coronavirus 2 (SARS‐CoV‐2) transmission in Hong Kong. SARS‐CoV‐2, like other respiratory viruses, is transmitted mainly via respiratory route in close proximity of or direct contact with asymptomatic or symptomatic individuals.

There is increasing evidence supporting the idea that SARS‐CoV‐2 is going to be an endemic disease in humans and hence co‐circulation with other common respiratory viruses in nature becomes inevitable.^2^ Previous studies have reported that the detection rate of other respiratory viruses among all SARS‐CoV‐2 positive cases was about 3.4% to 7.3%.^3^, ^4^ Such respiratory virus co‐infections may aggravate the severity of clinical manifestations and increase the rate of both those needing intensive care units and deaths of in‐patients.^5^ The prevalence of viral co‐infections is yet to receive enough attention. There is a need of prompt identification of patients' co‐infected with different respiratory viruses, thereby allowing timely treatments for this high‐risk group. Besides, there is also a need of monitoring the virus co‐circulation patterns in community for informing public health policy actions. Here, we report development of multiplex reverse transcription droplet digital PCR (RT‐ddPCR) assays for the detection of 15 different respiratory viruses, including adenovirus (Adv), human coronavirus (HCoV‐229E, HCoV‐HKU1, HCoV‐NL63, HCoV‐OC43), human enterovirus/rhinovirus (EV/RV), human metapneumovirus (HMPV), human parainfluenza virus types 1–4 (PIV1, PIV2, PIV3 and PIV4), influenza A virus, influenza B virus, respiratory syncytial virus (RSV) and SARS‐CoV‐2.

## 2 MATERIALS AND METHODS

### 2.1 Primer and probe sequences

Three assays with primer/probe sets targeting 15 common respiratory virus genes (Table S1) were developed: (1) Assay 1 detects influenza A virus M gene (Set 1a),^6^ PIV1 HN gene (Set 1b),^7^ SARS‐CoV‐2 RdRp gene (Set 1c),^8^ Adv hexon gene (Set 1d)^9^ and RSV N gene (Set 1e)^10^; (2) Assay 2 detects influenza B virus M gene (Set 2a),^6^ PIV2 HN gene (Set 2b),^10^ PIV3 HN gene (Set 2c),^10^ PIV4 nucleocapsid gene (Set 2d),^11^ EV/RV 5′‐UTR gene (Set 2e)^12^ and HMPV N gene (Set 2f)^13^; and (3) Assay 3 detects HCoV‐229E N gene (Set 3a),^14^ HCoV‐NL63 N gene (Set 3b),^14^ HCoV‐HKU1 N gene (Set 3c)^14^ and HCoV‐OC43 N gene (Set 3d).^14^ Several primer/probe sets (Sets 1d, 1e, 2b, 2c, 2e and 2f) were modified by reducing primer mismatches to our up‐to‐date virus sequence datasets, whilst others were obtained from previously published work as shown. The modified primer/probe sets were analysed in silico by an in‐house R program using the complete human virus sequences from Virus Pathogen Resources (viprbrc.org/), Influenza Research Database (fludb.org/) and National Centre for Biotechnology Information (ncbi.nlm.nih.gov/). The modified primers were considered optimal when they fulfilled all of the following criteria: (1) the last five bases from 3′ end perfectly match with their target sequences; (2) the last 10 bases from 3′ end do not have more than one mismatch to their targets and (3) the total number of mismatches does not exceed three bases. No modified probe should have more than one mismatch with the target sequences. All primers and probes were synthesized by Integrated DNA technologies with the label of 5′‐fluorophore (FAM or HEX), 3′‐Iowa Black FQ quencher and an internal ZEN quencher.

### 2.2 Archived specimens and viral nucleic acid extraction

Eighteen archived nasopharyngeal samples positive for Adv, EV, HCoV‐229E, HCoV‐HKU1, HCoV‐NL63, HCoV‐OC43, HMPV, PIV1, PIV2, PIV3, PIV4, RSV, RV, SARS‐CoV‐2, human influenza A virus (H1N1 or H3N2) or influenza B virus (Victoria [Vic] or Yamagata [Yam]) were used as control for assay optimization and evaluation. Viral RNA of these samples was extracted using QIAamp viral RNA mini kit (Qiagen) and the extracted RNA samples were stored at −80°C before further analyses.

### 2.3 The RT‐ddPCR assays for respiratory viruses

A total of 20 μl of standard PCR reaction mixture for all three assays was prepared by one‐step RT‐ddPCR Advanced Kit for Probes (Bio‐Rad) according to manufacturer's instructions. Each reaction consisted of 3 μl of diluted RNA, 5 μl of Supermix, 400 U of reverse transcriptase, 15 mmol/L dithiothreitol, 900 nmol/L for each primer and different probe concentrations as indicated (Assay 1: 250 nmol/L for Set 1a, 1b or 1c, 125 nmol/L for Set 1d and 50 nmol/L for Set 1e; Assay 2: 300 nmol/L for Set 2e, 250 nmol/L Set 2a or 2b and 125 nmol/L for Set 2c, 2d or 2f; Assay 3: 250 nmol/L for Set 3a or 3d, 125 nmol/L for Set 3c and 62.5 nmol/L for 3b). The droplet generation of PCR reaction mixture was performed by QX200™ droplet generator (Bio‐Rad) using DG8™ cartridge (Bio‐rad) and 70 μl of droplet generation oil (Bio‐rad). Emulsified reactions were then transferred to a 96‐well PCR plate for reaction to complete on T100™ thermocycler (Bio‐Rad) with following conditions: an initial reverse transcription step (42°C for 60 min and 95°C for 10 min), 40 cycles of PCR amplification (denaturation at 95°C for 30 s; annealing at 50°C for 60 s, extension at 65°C for 60 s), PCR inactivation at 98°C for 10 min and temporary storage at 4°C. The signals were detected by QX200™ droplet reader (Bio‐Rad) and analysed in fluorescence intensities by QuantaSoft™ Analysis Pro (Bio‐Rad). Reactions containing less than 10 000 droplets were disqualified for downstream analyses. The gating strategy for each viral target was optimized. No template control was included in all runs.

### 2.4 Preparation of plasmid standards

For evaluation of the respiratory virus panel, plasmid standards were prepared using RT‐ddPCR products and Zero Blunt® TOPO® PCR Cloning Kits (Invitrogen) based on manufacturer's instructions. The plasmid standards were confirmed by Sanger sequencing. All plasmids were digested by *EcoRI* (Thermo Scientific) following the manufacturer's instructions before subsequent experiments.

### 2.5 Evaluation of the respiratory virus panel

The cross‐reactivity and the limit of blank (LoB) was analysed by testing the mixture of the plasmid standards (10^4^ copies each, without the testing assay's targets) spiked in RNA extracted from negative human respiratory samples. The LoB for each assay was determined as 95th percentile of positive droplets for FAM and HEX signals in 20 replicates of negative reaction. Fifteen plasmid standards (Adv, HCoV‐229E, HCoV‐HKU1, HCoV‐NL63, HCoV‐OC43, HMPV, PIV1, PIV2, PIV3, PIV4, RSV, RV, SARS‐CoV‐2, H1 and Vic) were chosen to test the performance of the assays. Tenfold serial dilutions of the plasmid standards ranging from 10^5^ to 10^0^ were done to determine the dynamic range, intra‐assay and inter‐assay reproducibility. The intra‐assay reproducibility was determined by testing different concentrations in triplicates from the same run. The inter‐assay reproducibility was determined by testing two more replicates in another two different days within a week. Then, the dynamic range was determined by these five replicates from three different runs. The limit of quantification (LoQ) was determined by the lowest concentration of the plasmid standard of each target to be quantified in 16 replicates with CV ≤ 25%.^15^ The LoQ analyses were run for three different days (triplicates on day 1, five replicates on day 2 and eight replicates on day 3). The data generated for RT‐ddPCR performance were analysed using Prism 9.

### 2.6 Screening of retrospective samples for co‐infections

The co‐infection screening was done by testing 198 SARS‐CoV‐2 positive nasopharyngeal samples collected in two separate periods: 100 locally acquired cases from 1 December 2020 to 29 January 2021 (Wave 4 of Hong Kong), 98 imported cases from 23 November 2021 to 30 December 2021 (before Wave 5 of Hong Kong). These archived samples were all previously confirmed to be positive for SARS‐CoV‐2 by RT‐PCR.^16^

All samples were tested for all three assays. To reduce the testing cost and the screening time, in the first round of co‐infection screening three SARS‐CoV‐2 positive samples (each in 1 μl) were pooled for each assay. RNA samples in pooled reactions that were positive in the screening assays were then tested individually. The identities of the human respiratory viruses in the co‐infection cases were further confirmed by Sanger sequencing. The Ct values of SARS‐CoV‐2 single positive samples and those from co‐infection samples were compared by unpaired *t* test using Prism 9.

## 3 RESULTS

### 3.1 Assessment and evaluation of RT‐ddPCR assays for respiratory virus detections

**FIGURE 1:**
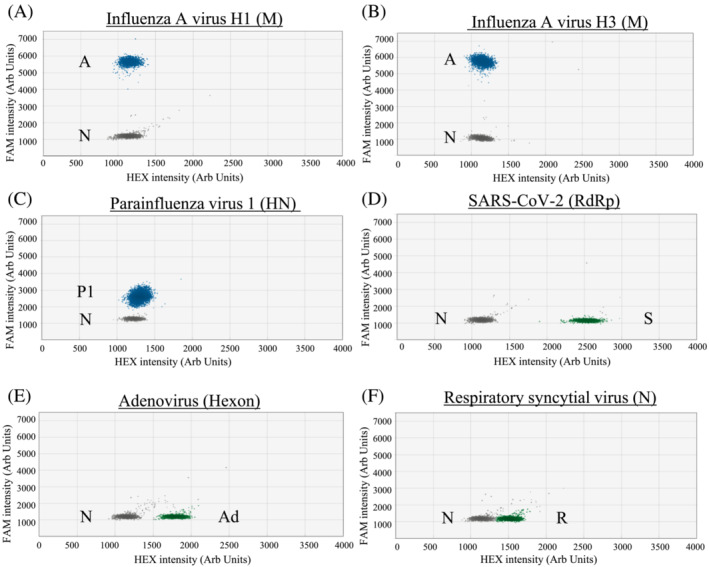
RT‐PCR droplets found positive for respiratory viruses in Assay 1. The *x* and *y* axes are the fluorescence amplitude in the HEX and FAM channels, respectively. Signals of RNA samples (up to 10^3^ copies per reaction) that are positive for a specific virus are shown as indicated (panels A–F). A, Influenza A virus (M gene); P1, PIV1 (HN gene); S, SARS‐CoV‐2 (RdRp gene); Ad, Adv (Hexon gene); R, RSV (N); N, Negative

**FIGURE 2:**
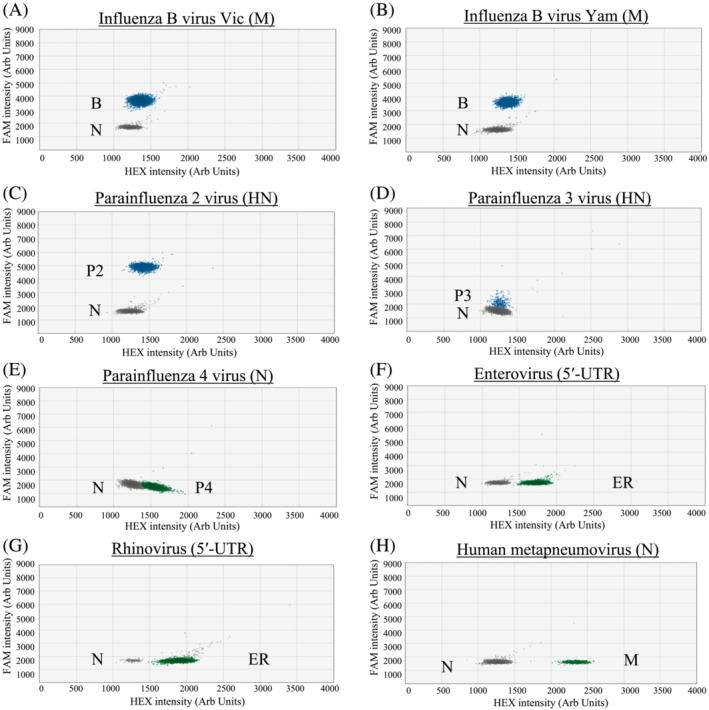
RT‐PCR droplets found positive for respiratory viruses in Assay 2. The *x* and *y* axes are the fluorescence amplitude in the HEX and FAM channels, respectively. Signals of RNA samples (up to 10^3^ copies per reaction) that are positive for a specific virus are shown as indicated (panels A–H). B, Influenza B virus (M gene); P2, PIV2 (HN gene); P3, PIV3 (HN gene); P4, PIV4 (N gene); ER, EV/RV (5′‐UTR); M, HMPV (N gene); N, Negative

**FIGURE 3:**
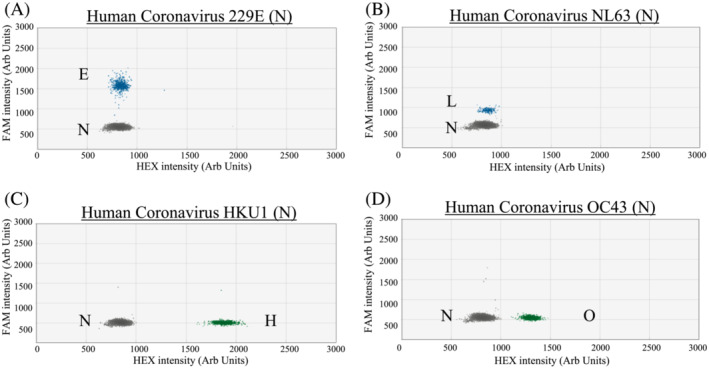
RT‐PCR droplets found positive for respiratory viruses in Assay 3. The *x* and *y* axes are the fluorescence amplitude in the HEX and FAM channels, respectively. Signals of RNA samples (up to 10^3^ copies per reaction) that are positive for a specific virus are shown as indicated (panels A–D). E, HCoV‐229E (N gene); L, HCoV‐NL63 (N gene); H, HCoV‐HKU1 (N gene); O, HCoV‐OC43 (N gene); N, Negative

Based on the designed criteria, the modified primer/probe sets have a good match to the great majority of our studied sequence targets (95.17% to 100%; Table S1). All archived positive control samples tested positive in the corresponding RT‐ddPCR and distinct positive signal clusters were observed in these reactions (Figures 1, 2, 3, blue or green droplets).

For single positive reactions in Assay 1, droplet positive for influenza A virus (Figure 1A for H1; Figure 1B for H3) and PIV1 (Figure 1C) had signals above 5,000 and below 3,500 fluorescence intensities, respectively, in the FAM channel. As for SARS‐CoV‐2 (Figure 1D), positive droplets had about 2,500 fluorescence intensities in the HEX channel. For Adv (Figure 1E) and RSV (Figure 1F), positive droplets were found to have about 1,750 and 1,500 fluorescence intensities, respectively, in the HEX channel. For single positive reactions in Assay 2, the positive droplets of influenza B virus (Figure 2A for Vic and Figure 2B for Yam) had signal intensity of about 3,600 fluorescence intensities in the FAM channel. The positive droplets of PIV2 (Figure 2C) were found to have >4,000 fluorescence intensities in the FAM channel. The positive droplets of PIV3 (Figure 2D) were found to have about 2,000 in the FAM channel. As for PIV4 (Figure 2E) and EV/RV (Figure 2F for EV and Figure 2G for RV), positive droplets had about 1,500 and 1,750 fluorescence intensities, respectively, in the HEX channel. For HMPV (Figure 2H), positive droplets had signal intensity of about 2250 fluorescence intensities in the HEX channel. For single positive reactions in Assay 3, positive droplets of HCoV‐229E (Figure 3A) and HCoV‐NL63 (Figure 3B) had signals at about 1,600 and 900 fluorescence intensities, respectively, in the FAM channel. For HCoV‐HKU1 (Figure 3C) and HCoV‐OC43 (Figure 3D) samples, positive droplets had signals at about 1,850 and 1,350 fluorescence intensities, respectively, in the HEX channel.

**FIGURE 4:**
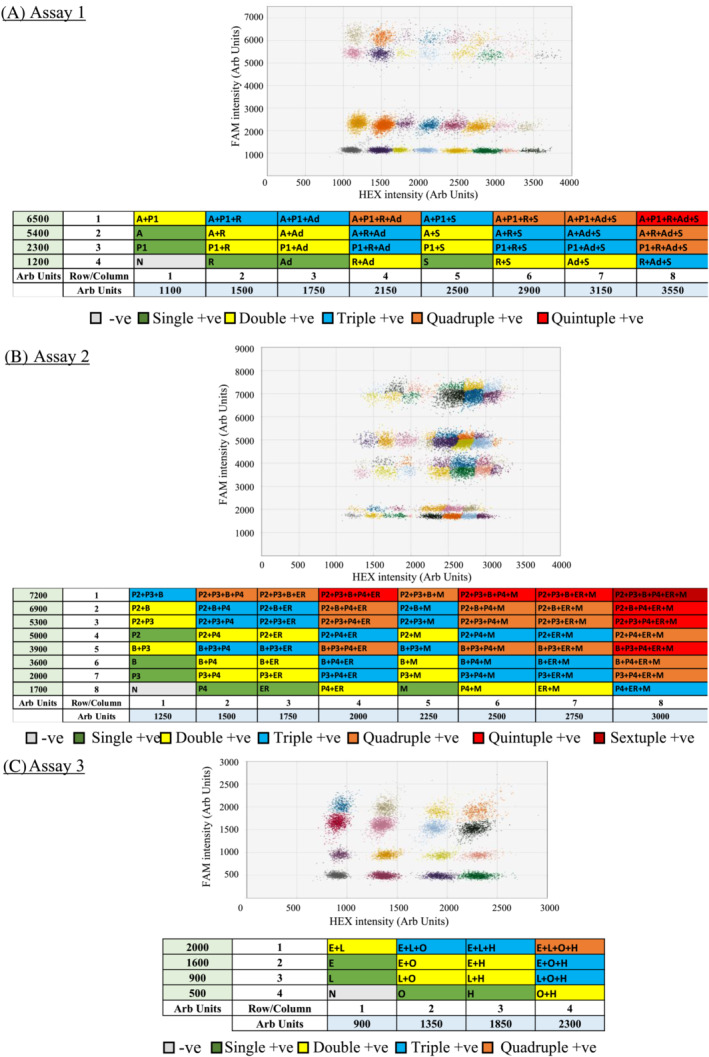
Classification of clusters for the assays: (A) Assay 1, (B) Assay 2 and (C) Assay 3 in 2D graphs where the *x* and *y* axes are the fluorescence amplitude in the HEX and FAM channels, respectively. Each graph showed the cluster distributions when all targets were added in each assay. Each target was added with not more than 10^3^ copies per reaction. The table below the 2D graphs demonstrated the location of clusters and corresponding amplitudes for the specific targets of each assay. Abbreviations: A, Influenza A virus (M gene); P1, PIV1 (HN gene); S, SARS‐CoV‐2 (RdRp gene); Ad, Adv (Hexon gene); R, RSV (N); B, Influenza B virus (M gene); P2, PIV2 (HN gene); P3, PIV3 (HN gene); P4, PIV4 (N gene); ER, EV/RV (5′‐UTR); M, HMPV (N gene); E, HCoV‐229E (N gene); L, HCoV‐NL63 (N gene); H, HCoV‐HKU1 (N gene); O, HCoV‐OC43 (N gene); N, Negative

When all positive controls were added to the same RT‐ddPCR reaction, more complex yet well‐separated cluster patterns emerged (Figure 4). Mixing different RNA samples into a single reaction do not affect droplet signal patterns specific for a particular viral target. In addition, droplet positive for more than one target formed expected patterns. The amplitude of signal clusters positive for two targets to be detected in the same channel were expected to be the sum of the net positive signal of each target (i.e., Signal_target‐background_) and the background signal (i.e., signal of negative droplet). For example, the double positive cluster of RSV+/Adv+ was located at about 2,150 fluorescence intensities in the HEX channel whereas the negative cluster was located at about 1,100 fluorescence intensities and the net amplitudes of RSV+ and Adv+ were 400 and 650, respectively (Figure 4A). By contrast, for targets that were independently detected by HEX and FAM channels, the magnitude of signals of double positive droplets in each channel was still identical to the single positive ones. For example, the positive cluster of influenza A+/RSV+ was at about 5,400 fluorescence intensities in the FAM channel and 1,500 fluorescence intensities in the HEX channel whereas the clusters of influenza A+ and RSV+ were at the same amplitude in the corresponding channel (Figure 4A).

All three assays were tested by human respiratory samples that were negative for the studied viruses. No cross‐reactivity was observed for any primer/probe sets, demonstrating that these assays were highly specific. Based on the results of cross‐reactivity study, the number of false positive droplets in negative reactions was determined. The LoB in FAM channel of Assays 1–3 was 4, 7 and 9 droplets, respectively. The LoB in HEX channel of Assays 1–3 was 5, 8 and 6 droplets, respectively. For each assay, multiple replicates were tested for intra‐assay (*N* = 3) and inter‐assay reproducibility (*N* = 5) (Table S2 for Assay 1, Table S3 for Assay 2 and Table S4 for Assay 3). All assays had a CV ≤ 25%, with lower concentration of standards tending to have a higher CV value of variability as expected. At least four orders of magnitude in dynamic range were displayed for PIV4 HN gene and HCoV‐OC43 N gene with *R* ^2^ more than 0.9682 (Figure S1). Other targets of respiratory virus panel showed five orders of magnitude in dynamic range with *R* ^2^ more than 0.9781. The LoQ was determined by testing low concentration of standards in 16 replicates. CV values of LoQ of all targets were less than 25% (Tables S2–S4), meeting the recommended standard for microbial detection.^15^ LoQ values of targets in copies per reaction were no more than 33 for Assay 1, 46 for Assay 2 and 45 for Assay 3.

### 3.2 Co‐infections with SARS‐CoV‐2 and other common respiratory viruses

**FIGURE 5:**
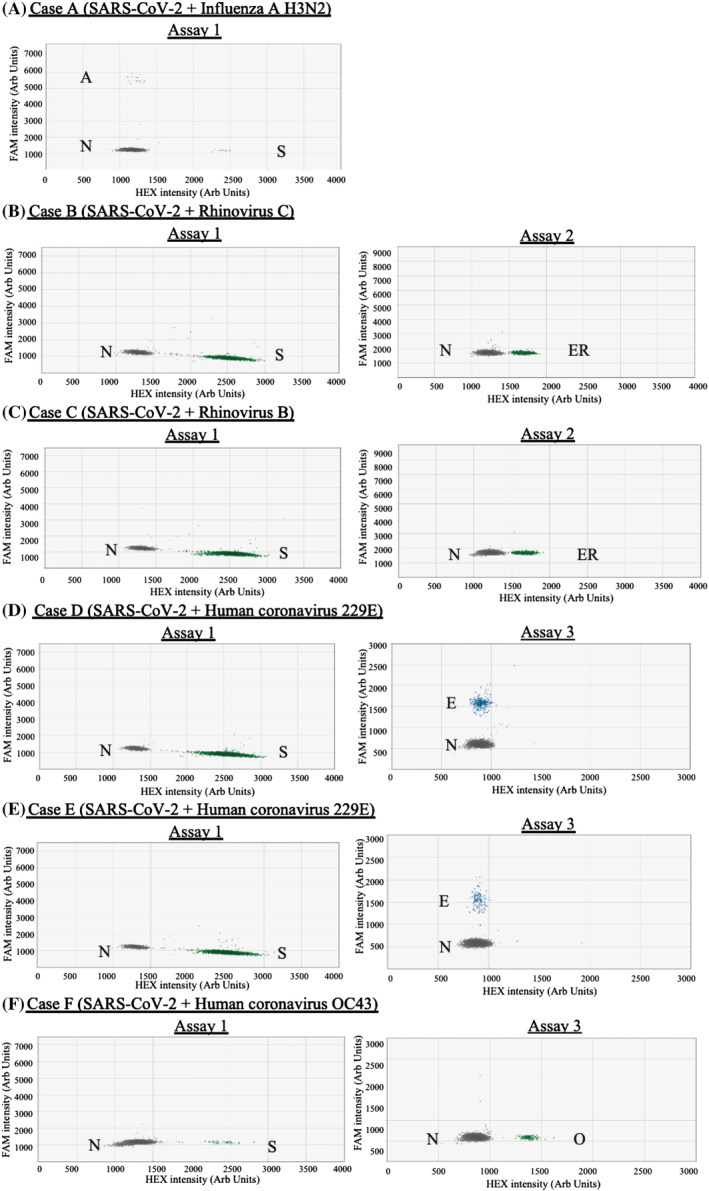
Classification of clusters for co‐infection samples: (A) case A; (B) Case B; (C) Case C; (D) Case D; (E) Case E and (F) Case F. The graph titles showed the reactions were done on the designated assay. The results were shown in 2D graphs where the *x* and *y* axes are the fluorescence amplitude in the HEX and FAM channels, respectively. Abbreviations: A, Influenza A virus (M); S, SARS‐CoV‐2 (RdRp); ER, EV/RV (5′‐UTR); E, HCoV‐229E (N); O, HCoV‐OC43 (N); N, Negative

The established assays were used to test 198 SARS‐CoV‐2 positive human specimen for respiratory virus co‐infection (Table 1). All samples were previously RT‐qPCR confirmed to be SARS‐CoV‐2 positive. Ct values of these samples in the SARS‐CoV‐2 test ranged from 12 to 37 and 14 to 37 for local and imported cases, respectively. The median of Ct values of local and imported cases were 21 and 25, respectively. A total of six SARS‐CoV‐2 positive samples from the imported cases (6.12%) were found to be co‐infected with other respiratory viruses (Figure 5; Case A with influenza A H3N2, Case B with Rhinovirus C, Case C with Rhinovirus B, Case D with HCoV‐229E, Case E with HCoV‐229E and Case F with HCoV‐OC43). The RT‐ddPCR amplicons from these co‐infected samples were further analysed by Sanger sequencing and the sequencing results were in concordance with the RT‐ddPCR findings. None of the locally acquired cases was found to be co‐infected with any target viruses besides SARS‐CoV‐2.

**TABLE 1:** Summary of SARS‐CoV‐2 positive samples for co‐infection detection

	Case definition	
	Local	Import
Collection period	2020/12/01 to 2021/01/29	2021/11/23 to 2021/12/30
Sample size	100	98
Ct value, median (range)	21 (12–37)	25 (14–37)
No. of co‐infection cases (%)	0 (0%)	6 (6.12%)

There was no significant difference between Ct values of the SARS‐CoV‐2 positive only and the co‐infection samples (*p* = 0.699, unpaired *t* test) (Figure S2). The amount of SARS‐CoV‐2 RNA and other respiratory viral RNA in the co‐infection samples ranged from 55 to 33 183 and 32 to 3465, respectively, copies per reaction (Table 2). These co‐infection cases were imported from Poland/Germany (Case A), Nepal (Case B), India (Case C), Switzerland/Finland (Case D), Ghana (Case E) and United Kingdom (Case F). Notably, carriers of these co‐infection cases had received at least two doses of COVID‐19 vaccine.

**TABLE 2:** Summary of import COVID‐19 cases with virus co‐infection

Case	Embarking country	Vaccination history	Sample collection date	Copies of SARS‐CoV‐2 per reaction	Co‐infected virus (copies per reaction)
A	Poland and Germany	Comirnaty × 2 (May and Jun 2021)	18/12/2021	5.46E+01	Influenza A, H3N2 (3.23E+01)
B	Nepal	Sinopharm × 2 (Jul and Aug 2021)	14/12/2021	1.05E+04	Rhinovirus C (1.33E+03)
C	India	Covishield × 2 (Jun and Sep 2021)	25/12/2021	2.21E+04	Rhinovirus B (3.47E+03)
D	Switzerland via Finland	Comirnaty × 2 (Both in May 2021)	7/12/2021	3.32E+04	HCoV‐229E (1.29E+03)
E	Ghana	Comirnaty × 2 (Both in Jun 2021)	22/12/2021	3.07E+04	HCoV‐229E (1.84E+02)
F	UK	Comirnaty × 3 (Mar, Apr and Nov 2021)	25/12/2021	6.09E+01	HCoV‐OC43 (2.42E+02)

## 4 DISCUSSION

In this study, we developed a panel of multiplex RT‐ddPCR assays for 15 common human respiratory viruses. Signals of positive droplets showed distinct clusters. Each of these assays have a broad dynamic range and a good assay reproducibility.

With the recent advances of RT‐ddPCR technology, viral nucleic acids can be detected by RT‐ddPCR assays using microfluidics. Each standard RT‐PCR reaction can be emulsified into >10,000 droplets. Thus, RT‐ddPCR can minimize PCR interference through partitions of potential PCR inhibitors and competitive amplicons. Moreover, determination of RT‐qPCR threshold is often set within the exponential phrase of the PCR reactions. The performance of RT‐ddPCR, like other end‐point PCRs, relies on the final point of amplification and is less affected by variability between efficiency of different PCR batches. In addition, the quantification of target sequences in droplet digital PCR is based on Poisson statistics.^17^ Unlike RT‐qPCR, RT‐ddPCR does not require a standard curve for absolute quantification. The whole process including the set‐up time and analysis time is approximately 6 h for 96 well‐plate. Further dilution is needed for absolute quantification of specimen that has the concentration of targets beyond the linear range of these assays.

Viral co‐infection potentially leads to increased morbidity and mortality.^4^ The prevalence of viral co‐infection among COVID‐19 positive cases was found to be about 7.3%. In the first 2 years of COVID‐19 pandemic, Hong Kong introduced a vast number of non‐pharmaceutical interventions to control COVID‐19.^18^ These control measures led to the suppression of other respiratory virus transmissions within Hong Kong, such as influenza A virus (chp.gov.hk/en/resources/29/304.html). By contrast, less stringent control measures were used in many overseas countries. The effectiveness of COVID‐19 mitigation measures in Hong Kong was evaluated by comparing two groups of COVID‐19 samples collected in this study, with 100 locally acquired cases during Wave 4 of Hong Kong and 98 imported cases before the start of Wave 5 in Hong Kong (Table 1). About 6.12% of the imported COVID‐19 cases were found to be infected by other respiratory viruses besides SARS‐CoV‐2, whereas none of the studied local COVID‐19 cases tested positive for another respiratory virus. The strict preventive measures, such as mandatory mask wearing, social distancing and prohibition of mass gathering applied in Hong Kong may have helped to control the transmission of common respiratory viruses. However, the lack of circulation of these respiratory viruses for an extended period might have reduced the herd immunity. It is possible that there will be a surge of virus co‐circulation once these non‐pharmaceutical control measures are lifted in Hong Kong.

This study has had its own limitations. The viral RNAs of the whole viruses isolated from cultured cells or clinical specimens were only used for the studies of specificity and cluster classifications. No undesirable cluster was observed and the subsequent experiments were followed by the use of plasmid standards. However, the use of plasmid standards for the evaluation of the respiratory virus panel has not validated the reverse transcription phrase of the reaction. It is possible that further verification may be needed when these assays are applied in other laboratories. Moreover, the probes for the detection of influenza A virus, influenza B viruses and EV/RV were designed to target the conserved regions of the viral genes. These assays were not able to perform subtyping or lineage differentiation for these viruses. Although the in silico evaluation has been performed for all the primer/probe sets (Table S1), only one archived specimen for each target was used for the evaluations and extra samples for each target are needed for a more inclusive validation of performances. On the other hand, we had no access to clinical data of the co‐infection cases. All co‐infected patients had received at least two doses of COVID‐19 vaccines before the infections. Based on the available data (Figure S2), there seems to be no significant difference (*p* = 0.699) between Ct values of SARS‐CoV‐2 and COVID‐19‐only and the co‐infection samples. Previous studies indicated that Omicron variant of SARS‐CoV‐2 may lead to a less severe clinical manifestation in comparison to Delta variant.^19^ The outcome of viral co‐infection with different SARS‐CoV‐2 variants still need more investigation, a comprehensive study of disease severity in co‐infection patients can be done with more information of clinical diagnosis and prognosis. Nevertheless, sequential infections with other respiratory viruses are shown to be capable of modulating the replicative capacity of SARS‐CoV‐2 in the upper respiratory tract.^20^ Recent studies have further showed that a primary influenza A virus infection can elevate the expression of ACE2 in humans, suggesting that the co‐infection of COVID‐19 and influenza A may lead to an aggravated clinical manifestation.^21^ Previous study showed that co‐infections of Adv or RSV with SARS‐CoV‐2 had no differences in in‐patient outcomes compared with SARS‐CoV‐2 mono‐infections.^22^ Analysing serial samples of co‐infected patients might help to better understand the viral dynamics or interferences during virus co‐infection. Besides, all specimens tested in this study were nasopharyngeal swab samples. The results can reflect the virus replication events in the upper respiratory tract. Other specimen types like tracheal aspirate may help to study the effect of virus co‐infection on viral replication in the lower respiratory tract.

Overall, this work has developed a panel of three multiplex RT‐ddPCR assays for detection of 15 common human respiratory viruses. This tool is handy for high throughput screening for virus co‐infection. A timely and accurate detection of SARS‐CoV‐2 and other respiratory viruses can allow prompt clinical treatment and inform timely public health policy.

## CONFLICT OF INTEREST

The authors declare that they have no competing interests.

## AUTHOR CONTRIBUTIONS

**Nathaniel Leong:** Formal analysis; investigation; methodology; visualization; writing‐original draft; writing‐review and editing. **Haogao Gu:** Data curation; investigation; methodology; software. **Daisy Ng:** Formal analysis; investigation; methodology. **Lydia Chang:** Data curation; investigation; methodology. **Pavithra Krishnan:** Data curation; investigation; methodology. **Samuel Cheng:** Data curation; investigation; methodology. **Malik Peiris:** Conceptualization; formal analysis; resources; supervision. **Leo Poon:** Conceptualization; funding acquisition; project administration; supervision; writing‐original draft; writing‐review and editing.

### PEER REVIEW

The peer review history for this article is available at https://publons.com/publon/10.1111/irv.13084.

## Supporting information


**Table S1.** Primers and probes used in this study.
**Table S2.** Intra‐/inter‐assay reproducibility and limit of quantification of Assay 1.
**Table S3.** Intra‐/inter‐assay reproducibility and limit of quantification of Assay 2
**Table S4.** Intra‐/inter‐assay reproducibility and limit of quantification of Assay 3

**Figure S1.** Dynamic range were determined by the plasmid standards of targets for A) Assay 1, B) Assay 2 and C) Assay 3. The targets of the reaction, the slope, the intercept and the R2 values were written as the legend of each graph. Five replicates from three runs were done for each dilution factor.
**Figure S2.** Comparison of Ct values between SARS‐CoV‐2 samples and co‐infection samples. Unpaired t‐test was used for the comparison of Ct values between the SARS‐CoV‐2 samples and co‐infection samples collected from the import cases before wave 5 of Hong Kong. Ct values were determined by RT‐qPCR of SARS‐CoV‐2. ns: non‐significant.

## Data Availability

The data that support the findings of this study are available from the corresponding author upon request.
