# Supplementary material for: Development of multiplex RT‐ddPCR assays for detection of SARS‐CoV‐2 and other common respiratory virus infections
Source: Influenza Other Respir Viruses. 2022 Dec 14;17(1):e13084. doi: 10.1111/irv.13084 (PMC9835441; doi:10.1111/irv.13084)
Supplement: Supplementary file 1 — Table S1. Primers and probes used in this study. Table S2. Intra‐/inter‐assay reproducibility and limit of quantification of Assay 1. Table S3. Intra‐/inter‐assay reproducibility and limit of quantification of Assay 2 Table S4. Intra‐/inter‐assay reproducibility and limit of quantification of Assay 3 [file IRV-17-0-s001.docx]

**Supplemental Tables**

**Supplemental Table 1: Primers and probes used in this study.**

**Supplemental Table 2: Intra-/inter-assay reproducibility and limit of quantification of Assay 1.**

**
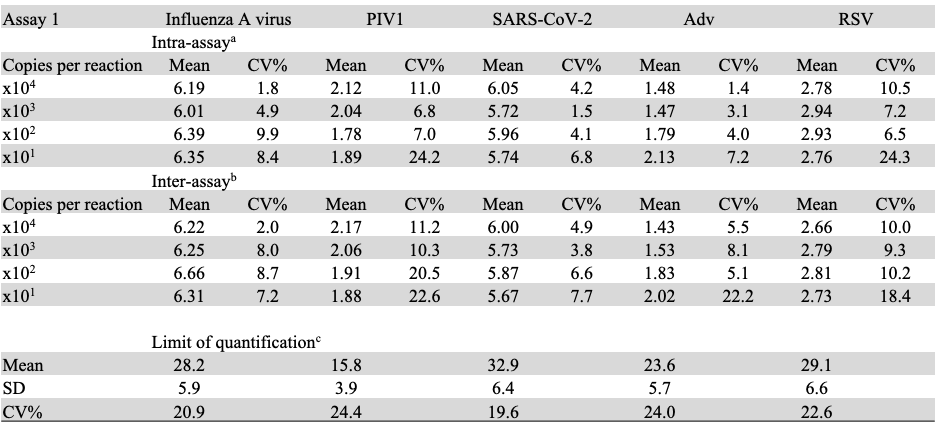
**

^a No. of copies per reaction in triplicate on same day^

^b No. of copies per reaction in 5 replicates on three different days^

^c Average of sixteen replicates, in copies per reaction^

**Supplemental Table 3: Intra-/inter-assay reproducibility and limit of quantification of Assay 2**

**
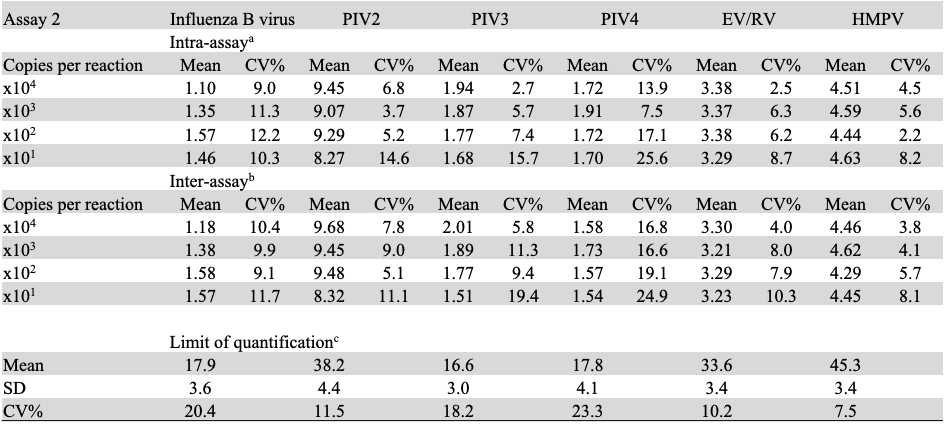
**

^a No. of copies per reaction in triplicate on same day^

^b No. of copies per reaction in 5 replicates on three different days^

^c Average of sixteen replicates, in copies per reaction^

**Supplemental Table 4: Intra-/inter-assay reproducibility and limit of quantification of Assay 3**


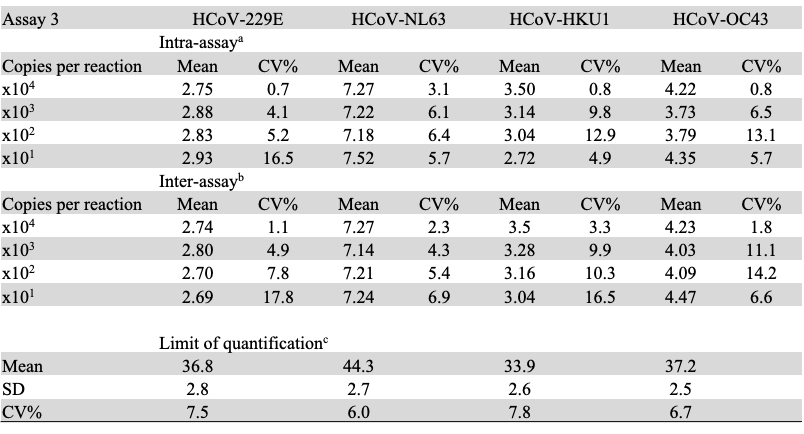


^a No. of copies per reaction in triplicate on same day^

^b No. of copies per reaction in 5 replicates on three different days^

^c Average of sixteen replicates, in copies per reaction^
