# Supplementary figures and images for: Development of multiplex RT‐ddPCR assays for detection of SARS‐CoV‐2 and other common respiratory virus infections
Source: Influenza Other Respir Viruses. 2022 Dec 14;17(1):e13084. doi: 10.1111/irv.13084 (PMC9835441; doi:10.1111/irv.13084)

## Slide 1
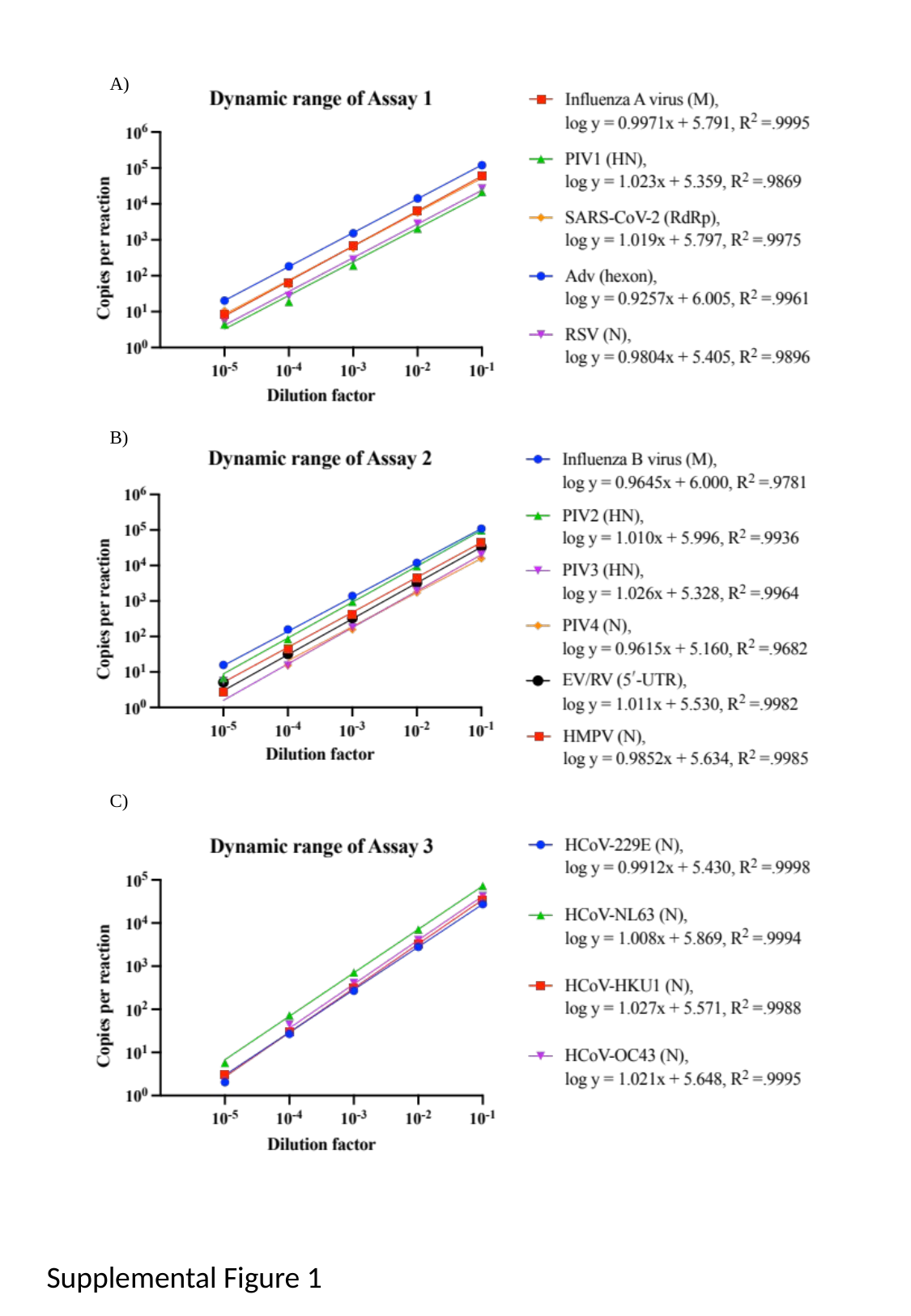

A)
B)
C)
Supplemental Figure 1

## Slide 2
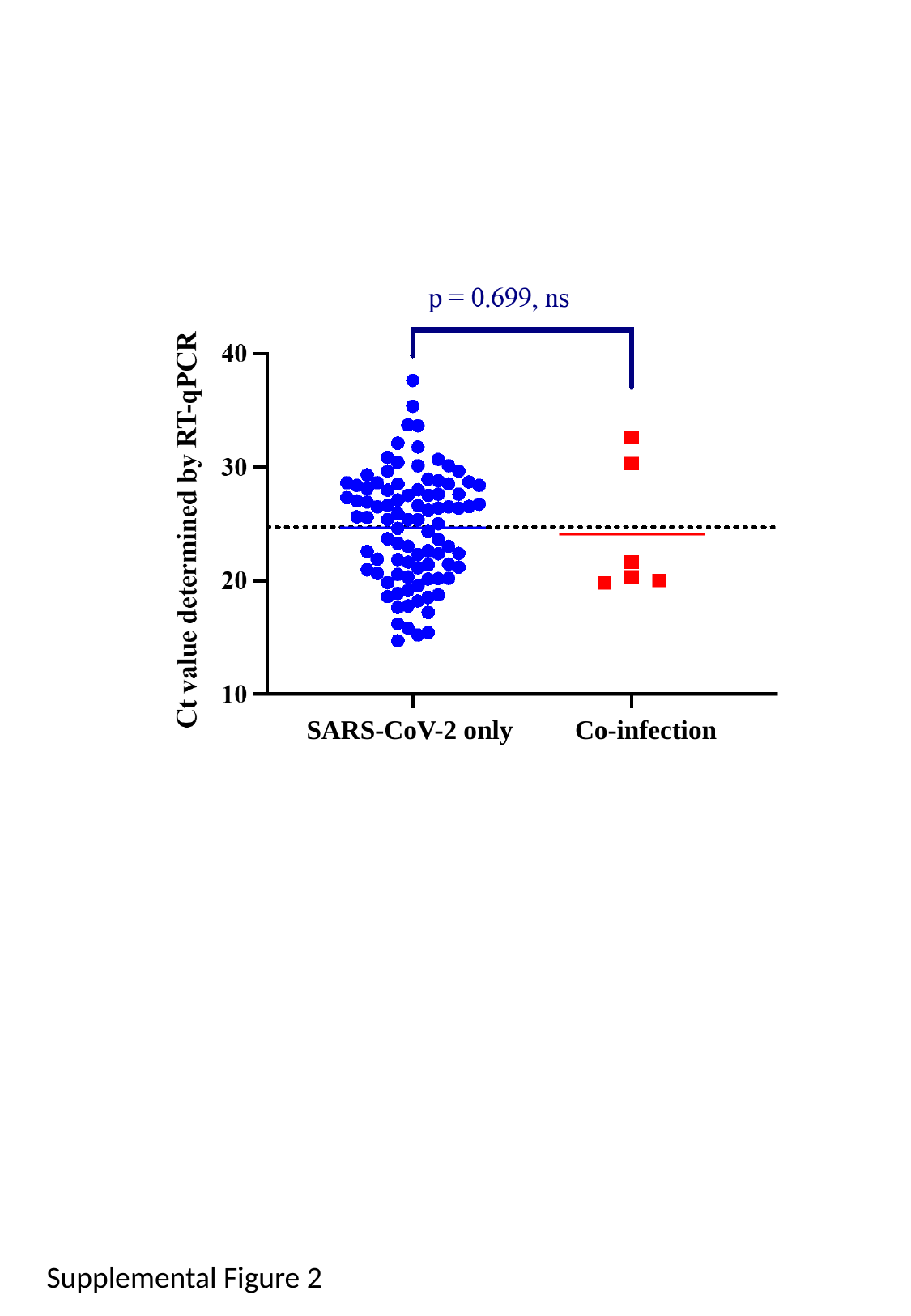

SARS-CoV-2 only
Co-infection
Supplemental Figure 2

Supplement: Supplementary file 2 — Figure S1. Dynamic range were determined by the plasmid standards of targets for A) Assay 1, B) Assay 2 and C) Assay 3. The targets of the reaction, the slope, the intercept and the R2 values were written as the legend of each graph. Five replicates from three runs were done for each dilution factor. Figure S2. Comparison of Ct values between SARS‐CoV‐2 samples and co‐infection samples. Unpaired t‐test was used for the comparison of Ct values between the SARS‐CoV‐2 samples and co‐infection samples collected from the import cases before wave 5 of Hong Kong. Ct values were determined by RT‐qPCR of SARS‐CoV‐2. ns: non‐significant. [file IRV-17-0-s002.pptx]
